# MicroRNA Expression Profiling in PBMCs: A Potential Diagnostic Biomarker of Chronic Hepatitis C

**DOI:** 10.1155/2014/367157

**Published:** 2014-11-18

**Authors:** Chiu-Chun Chang, Chun-Che Lin, Wan-Ling Hsieh, Hsin-Wu Lai, Chia-Hsun Tsai, Ya-Wen Cheng

**Affiliations:** ^1^Institute of Medicine, Chung Shan Medical University, Taichung, Taiwan; ^2^Division of Gastroenterology, Department of Internal Medicine, Chung Shan Medical University Hospital, Taichung, Taiwan; ^3^School of Medicine, Chung Shan Medical University, Taichung, Taiwan; ^4^Department of Pathology, Chung Shan Medical University, Taichung, Taiwan; ^5^Graduate Institute of Cancer Biology and Drug Discovery, College of Medical Science and Technology, Taipei Medical University, Taipei 115, Taiwan

## Abstract

The expression levels of miR-16, miR-193b, miR-199a, miR-222, and miR-324 in PBMCs were significantly higher in CHC patients compared with healthy controls and significantly different between CHC patients with HCV genotype 1 (GT-1) and non-genotype-1 (non-GT-1). Multivariate logistic regression analysis also showed that patients with high expression levels of the six target miRNAs had an approximately 7.202-fold risk of CHC compared with those with low expression levels of the target miRNAs. We concluded that the expression levels of miR-16, miR-193b, miR-199a, miR-222, and miR-324 target miRNAs in PBMCs of CHC may act as significant risk biomarkers for the development of CHC.

## 1. Introduction

Hepatitis C virus (HCV) infection affects more than 3% of the world's population [1]. HCV infection frequently induces chronic liver disease, ranging from chronic hepatitis C (CHC) to liver cirrhosis and hepatocellular carcinoma (HCC) [1]. HCV can interact with host cells to modulate cell survival signaling, alter gene expression, and induce cell transformation [2].

miRNAs are endogenous, small (approximately 22-nucleotide) noncoding RNAs that downregulate gene expression [3]. Some miRNAs are produced at high concentrations within cells in a tissue-specific manner [4, 5], and such miRNAs have recently been reported to be remarkably stable in plasma [4, 6]. miRNA-122 (miR-122) is a highly abundant, liver-expressed microRNA [7, 8]. Research has shown that miR-199a directly regulates HCV replication and suggested that it may serve as a novel antiviral therapy [9]. In addition, a previous computational study of HCV signaling pathways suggested a critical role for miRNAs in the replication, propagation, and latency of viruses in the host cell [10]. Specifically, the study revealed that miR-122, miR-320, and miR-191 were downregulated and that miR-215, miR-16, miR-26, miR-130, miR-199, and miR-155 were upregulated in HCV infected cells [11]. Based on these, findings suggest that miRNAs have the potential to become novel drug targets in viral-induced infectious or malignant diseases [11].

An ideal biomarker of CHC should be released into the systemic circulation or other body fluids, where it can be detected in a blood-based assay or assay of another accessible body fluid [12–14]. Peripheral blood mononuclear cells (PBMCs) have been reported to play an important role in HCV progression [15]. Induction of PBMC-miR-155 was found in patients with chronic HCV infection [16]. In addition, HCV core induces STAT3 as a result of the alteration of inflammatory response by antigen-presenting cells via an IL-6 autocrine pathway [17]. There are several disease-associated miRNAs in PBMC reported in previous studies [18, 19]. Thus, expression levels of miRNAs in PBMC may correlate to HCV infection and treatment outcome to anti-HCV therapy and potentially serve as noninvasive markers.

In this study, we analyzed the expression profiles of miRNAs in PBMCs of CHC patients and healthy controls. Specific expression patterns found in the CHC patients relative to the healthy controls suggested that miRNA activities may potentially contribute to the pathobiology of HCV infection and could be useful diagnostic biomarkers.

## 2. Material and Methods

### 2.1. Study Population

Peripheral blood samples were collected from 91 CHC patients who were treated with IFN alpha plus ribavirin at the Department of Internal Medicine, Chung Shan Medical University Hospital, and from 48 healthy controls, between October 2010 and December 2012. Informed written consent was obtained from all of the subjects and/or guardians prior to the use of their blood specimens. The acquisition of the samples and their subsequent examination were approved by the Institutional Review Board of Chung Shan Medical University. None of the participants had a previous history of cancer. The demographic and clinical data of the patients at the time of the sample collection are summarized in Table 1.

All participants gave their informed consent, and the following criteria were met. (i) All patients had an established diagnosis of CHC. (ii) All patients had an absence of another cause of chronic liver disease and an absence of viral coinfection. (iii) All patients received treatment with either PEG-IFN-*α*2b (Viraferonpeg; Schering Plough) at a dose of 1.5 *μ*g/kg of body weight/week and weight-based ribavirin at a dose of 800 to 1,200 mg/day (Rebetol; Schering Plough) or PEG-IFN-*α*2a at a dose of 180 *μ*g/week (Pegasys; Roche) and weight-based ribavirin at 1,000 to 1,200 mg/day (Copegus; Roche). The duration of treatment was 48 weeks for the patients with HCV genotypes 1 and 4 and 24 weeks for those with genotypes 2 and 3. For patients with HCV genotypes 1 and 4, if the HCV RNA was detectable at week 24, the treatment was stopped. For those with HCV genotype 1, if the HCV RNA decreased less than 2 log10 from the baseline to week 12 of therapy, treatment was stopped. (iv) All individuals with CHC enrolled in this study had known HCV genotypes. (v) Blood samples were collected before and after IFN alpha plus ribavirin treatment. Tumor tissues were collected from hepatocellular carcinoma patients infected with HCV to be used to compare the miR-122 expression.

### 2.2. MicroRNA Profiling

Low-molecular weight RNAs of less than 200 nt were extracted from the PBMCs of 10 CHC patients and a reference pool of 10 paired healthy controls. The total RNA was extracted from the PBMCs of the HCV patients using TRIzol (Invitrogen, Carlsbad, CA), where the TRIzol was supplemented with 0.2 mL of chloroform per 1 mL of reagent, mixed, and centrifuged (15 min; 12,000 g; 4°C). The supernatant was retained. To pellet the RNA, 0.5 mL of isopropanol (per 1 mL of reagent) was added and the mixture was incubated for 10 min at room temperature, followed by centrifugation (15 min; 12,000 g; 4°C). The pellet was washed once in 0.5 mL of 75% ethanol. The final RNA products were quantified by absorbance measurements at 260 nm (A260) and 280 nm (A280). The A260/A280 values were higher than 1.6 for all of the samples. A measure of 5 *μ*L of RNA was reverse-transcribed using the TaqMan miRNA reverse-transcription kit. The miRNA profiles were analyzed by MegaplexTM Pools for MicroRNA Expression Assays (MegaplexTM RT Primers, Human Pools A and B; Applied Biosystems, Foster City, CA). The detailed microRNA list is shown on the following website: http://www.lifetechnologies.com/order/catalog/product/4444745/.

### 2.3. Real-Time PCR-Based Detection of MicroRNAs

The detailed methods of isolating short RNAs and the reverse-transcription reaction were the same as those described in Section 2.2. The expression of mature miRNA was detected by a TaqMan miRNA assay according to the instructions of the manufacturer (Applied Biosystems) and normalized using the 2 − ΔΔCT-method relative to U6-snRNA. The CT (cycle threshold) was inversely proportional to the amount of target nucleic acid in the sample, and all of the TaqMan-PCRs were performed in triplicate. The definitions of the high and low expressions of the microRNA were dependent on the mean value of all patients. The expression levels higher than the mean were defined as high expressions, while the expression levels lower than the mean were defined as low expressions.

### 2.4. Statistical Analysis

All data were analyzed using the Statistical Package for the Social Sciences version 13.0 software (SPSS Inc., Chicago, IL). The Chi-square test (*χ* ^2^ test) was used to compare the expression of miRNAs and clinicopathological parameters. A probability of less than 0.05 was considered statistically significant. To determine whether the expression of the miRNAs could be used as an independent risk factor for HCV infection, a multiple unconditional logistic model was used to obtain the adjusted odds ratios (ORs) for the difference in gender, age, and BMI, and a corresponding 95% CI was used for the *T*-score after adjusting for the effect of potential confounding factors. The factors used in the multivariate analysis were analyzed by univariate analysis first. If the *P* value was less than 0.05, they were subjected to multivariate analysis.

## 3. Results

### 3.1. miR-122 Was Downregulated in the PBMCs of CHC Patients

**Figure 1:**
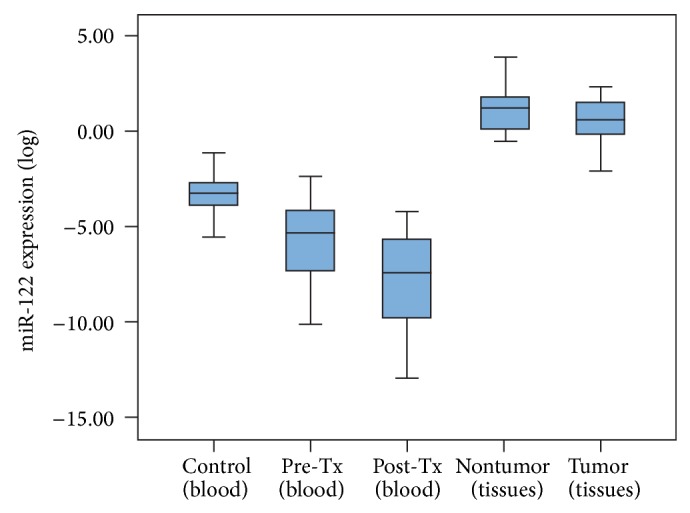
The expression levels of miR-122 in PMBCs of CHC patients and healthy controls were significantly lower than in tumor and nontumor tissues of liver cancer. The miR-122 expression level was detected by real-time PCR.

Previous reports showed that the expression levels of miR-122 were decreased in the liver tissues of CHC patients [7, 8, 11, 20]. To understand whether the expression levels of miRNAs in PBMCs could be used as biomarkers for CHC diagnosis, the expression of miR-122 was used as a control. As shown in Figure 1, the expression levels of miR-122 in the PBMCs of the CHC patients were significantly lower than in the healthy controls (*P* < 0.001). We used 20 paired tumor and nontumor HCC tissues to confirm the results from the PBMCs of the CHC patients (Figure 1). The expression of miR-122 was higher in the normal tissues than in the tumor tissues of the CHC patients. The results are consistent with previous reports [8, 9, 12, 16]. We also found that the expression levels of miR-122 in the tumor and the normal tissues of the HCC patients were significantly higher than in the PBMCs of the CHC patients and that the expression of miR-122 in the PBMCs of the CHC patients was significantly lower than in the healthy controls.

### 3.2. Expression Levels of MicroRNAs Were Associated with HCV Genotypes and Differed between the CHC Group and the Healthy Controls

**Figure 2:**
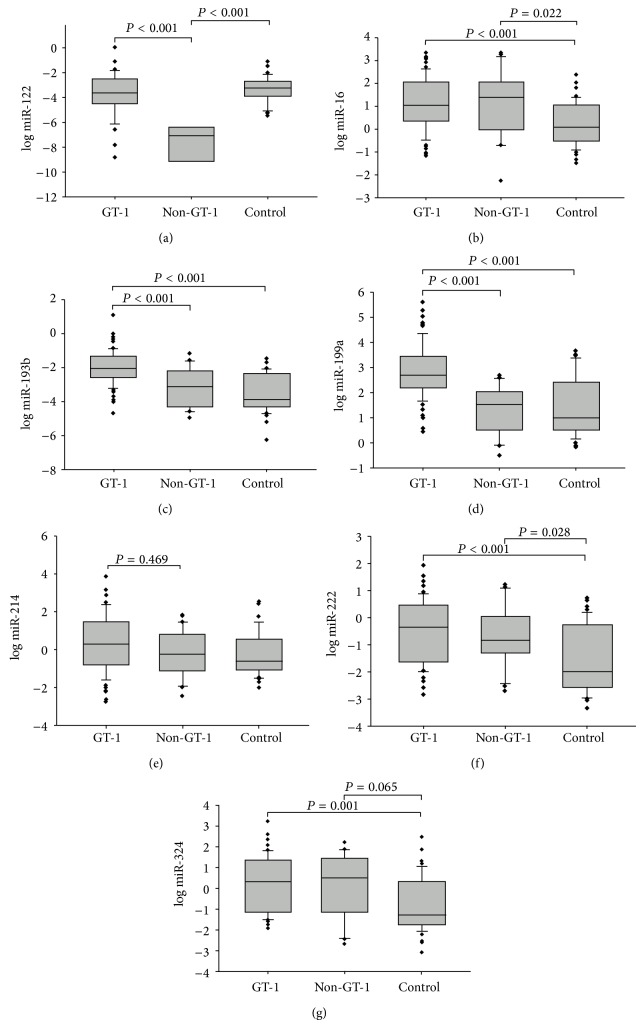
Target miRNAs expression in PBMCs of CHC patients with different HCV genotype and healthy control groups.

We analyzed miRNA expression profiles in the PBMCs of 10 CHC patients and 10 paired healthy controls using a miRNAs array. We identified miRNAs that were significantly differentially expressed in the CHC patients and the control group. The results of a global miRNA expression analysis of the PBMCs of the 91 CHC patients compared with the 48 healthy donors are shown in Table 2. We selected five target miRNAs (miR-199a-3p, miR-324, miR-16, miR-222, and miR-214) that had been studied in HCC tumor tissues for further analysis. To determine whether the expression levels of the miRNAs could be used as diagnostic markers of CHC, their expression levels in the PBMCs of the CHC patients and the healthy controls were analyzed by real-time RT-PCR. As shown in Table 3, the expression levels of miR-122, miR-16, miR-193b, miR-199a, miR-222, and miR-324 were significantly higher in the PBMCs of the CHC patients compared with the healthy controls (miR-16, *P* = 0.001; miR-122, *P* < 0.001; miR-193b, *P* < 0.001; miR-199a, *P* < 0.001; miR-222, *P* < 0.001; and miR-324, *P* = 0.001). No significant difference was found in the expression of miR-214 between the CHC group and the healthy controls (*P* = 0.469). In addition, the expression levels of these six miRNAs were significantly different between the CHC patients with a HCV genotype 1 and a non-genotype-1 (miR-16, *P* = 0.001; miR-122, *P* < 0.001; miR-193b, *P* < 0.001; miR-199a, *P* < 0.001; miR-222, *P* < 0.001; and miR-324, *P* = 0.001; Figure 2).

### 3.3. Expression of Six MicroRNAs Could Be Used as Biomarkers of CHC

To further confirm whether the changes in the expression levels of these miRNAs could be used as a useful biomarker in HCV infection, we analyzed the association of the miRNA levels and clinical factors (such as ALT, AST, and liver cirrhosis) of the CHC patients. As shown in Table 4, the expression levels of the miR-16, miR-122, miR-193b, miR-199a-3p, miR-222, and miR-324-3p were significantly correlated with the changes in the ALT and AST of the CHC patients, but no correlation between miR-214 and these clinical factors was found (Table 4). No correlation of target miRNAs and liver cirrhosis was found in this study. Multivariate logistic regression analysis was used to verify whether the expression levels of the miRNAs acted as independent risk biomarkers for CHC (Table 5). The expression levels of the miRNAs were approximately normally distributed after log transformation. Among the variables studied were miRNA expression levels, age, gender, and BMI. The data showed that those with high expression levels of the six target miRNAs had an approximately 7.202-fold risk of CHC compared with the those with low expression levels of these miRNAs (95% confidence interval (CI) = 2.014–25.752, *P* = 0.002). The odds ratios of the other variables were gender (OR = 0.227, 95% CI = 0.080–0.648, and *P* = 0.006) and age (OR = 1.182, 95% CI = 1.106–1.262, and *P* < 0.001). The OR of BMI did not reach statistical significance (OR = 2.090, 95% CI = 0.394–11.074, and *P* = 0.386; Table 4). Thus, the expression levels of six miRNAs in the PBMCs of CHC patients acted as significant risk biomarkers for the development of CHC.

## 4. Discussion

Several studies have demonstrated a relationship between HCV and host miRNA, especially miR-122, the most abundant miRNA in the liver [7, 8, 11, 20]. Previous reports provided evidence that after knockdown miR-122 processing machinery in culture cells significantly reduced HCV viral RNA [21]. In the present study, the expression of miR-122 was not correlated with clinical parameters of CHC, including the viral load (see Supplemental Table  1 in Supplementary Material available online at http://dx.doi.org/10.1155/2014/367157). Thus, we compared the expression levels of miR-122 in the liver tissues and PBMCs of the CHC patients. The results showed that the expression level of miR-122 was significantly lower in the PBMCs of the CHC patients than in the liver tissues (Figure 1). In addition, the level of expression of miR-122 in the PBMCs of the CHC patients was significantly lower than in the healthy controls. Thus, we consider that the expression level of miR-122 in PBMCs could be used as a diagnosis marker of CHC but not to monitor amplification of the virus.

Changes of microRNAs in the liver have been reported in disease processes such as hepatocarcinogenesis and liver fibrosis [22–26]. However, detecting these biomarkers using tissues was difficult to apply in clinical practice to monitor the disease. Detection of miRNAs in peripheral blood, either cell-associated or cell-free, holds great potential for noninvasive and real-time molecular based monitoring of diagnosis and treatment effects in disease. Extracellular miRNAs are remarkably stable in the circulation [27–30]. Previous reports showed that some miRNAs were released from their cells of origin and could be captured in various extracellular fluids; numerous studies began investigating whether tissue- and disease-specific miRNA signatures were also reflected in fluids such as blood, urine, spinal fluid, or saliva [27–29, 31, 32]. As a result of cellular damage/tissue injury, such as in acute myocardial infarction (MI) [33–35], atherosclerosis [36], non–small cell lung cancer [37], neurodegenerative diseases [38–41], skin fibrosis [42], and osteoarthritis [43], miRNA expression can change in the blood. Thus, circulating miRNAs are attractive candidates for disease monitoring to serve as valuable prognostic indicators of disease progression or resolution.

There is however only limited information about their detection in blood and their correlation with histological disease severity in patients with CHC. Previous studies have elected to use miRNA from serum instead of miRNA from exosome as the candidate for diagnosing diseases [28, 44–47]. Nathwani et al. mentioned that variations in the concentration of miR-122 in serum or plasma tend to be more specific for liver diseases than ALT and AST. This is because miR-122 is almost exclusively expressed in the liver, whereas ALT and AST originate from skeletal muscles and other tissues; therefore their diagnostic value is low [48]. In the present study, we also found that the expression levels of miR-16, miR-122, miR-193b, miR-199a-3p, miR-222, and miR-324-3p were significantly correlated with the changes in the ALT and AST of the CHC patients (Table 4). In addition, the present study revealed that those with high expression levels of the target miRNAs had an approximately 7.202-fold risk of CHC compared with those with low expression levels of six target miRNAs (95% confidence interval (CI) = 2.014–25.752, *P* = 0.002). Thus, we consider that the expression levels of these six miRNAs in the PBMCs of CHC patients act as significant risk biomarkers for the development of CHC.

A previous report showed that after full-length HCV was transfected into HepG2 cells, levels of miR-193b increased, whereas those of the predicted downstream target Mcl-1 gene decreased compared with a parental control [49]. Peng et al. (2009) carried out a computational study of HCV associated miRNA-mRNA regulatory modes in human livers. They found that miR-122, miR-320, and miR-191 were downregulated, whereas miR-215, miR-16, miR-26, miR-130, miR-199, and miR-155 were upregulated [11]. Murakami et al. (2009) demonstrated that miR-199a^*^ can control HCV viral replication [9]. The current study showed that levels of miR-193b and miR-199a-3p were significantly higher in the PBLs of HCV patients than in the control group. In addition, we found that those with high levels of expression of miR-193b and miR-199a-3p had an approximately 6.545-fold risk of CHC compared with those with low levels of expression of miR-193b and miR-199a-3p (95% CI = 2.180–19.651, *P* = 0.001; Supplemental Table 2). Thus, we suggest that the expression level of miRNAs may serve as a useful biomarker for HCV infection.

In conclusion, we found specific expression patterns of miRNAs in CHC patients compared with healthy controls, suggesting the potential contribution of miRNA activities to the pathobiology of HCV infection. Changes in the levels of miRNAs in PBMCs in CHC may act as significant diagnosis biomarkers for CHC.

## Supplementary Material

In the present study, the expression of miR-122 was not correlated with clinical parameters of CHC, including the viral load (Supplemental Table 1). In addition, we found that those with high levels of expression of miR-193b and miR-199a-3p had an approximately 6.545-fold risk of CHC compared with those with low levels of expression of miR-193b and miR-199a-3p (95% CI =2.180–19.651, p=0.001; Supplemental Table 2).

## Figures and Tables

**Table 1:** The clinical information of CHC patients in this study.

Parameters	
Gender, male/female	60/31 (2 : 1)
Age (years)	55.1 ± 10.6
BMI	24.5 ± 3.4
HCV RNA (log⁡IU/mL)	6.28 ± 0.93
AST	82.7 ± 75.5
ALT	121.3 ± 97.9
*γ*GT	88.6 ± 134.1
Cr	0.79 ± 0.19
Cholesterol	163.1 ± 30.1
TG	136.0 ± 147.8
Glucose	123.7 ± 47.4
WBC	5878.8 ± 1580.8
Hb	14.9 ± 1.5
PLT: >15000/<15000	53/38
Genotype-1/non-1	71/20

CHC: chronic hepatitis C; BMI: body mass index; AST: aspartate aminotransferase; ALT: alanine aminotransferase; Cr: creatinine; TG: triglyceride; PLT: platelet.

**Table 2:** Up- and downexpressed microRNAs in PBMCs of CHC patients compared with the healthy donors.

MicroRNAs	Endogenous control			
	RUN44		U6B	
	RQ^*^	log⁡RQ	RQ^*^	log⁡RQ
Downregulated miRNAs				
miR-330-3p	0.01	−1.96	0	−3.82
miR-371-3p	0.02	−1.66	0	−3.53
miR-501-5p	0.04	−1.4	0	−3
miR-636	0.04	−1.37	0	−3
miR-214	0.08	−1.08	0	−3
miR-886-3p	0.09	−1.07	0	−3
miR-548c-5p	0.11	−0.97	0	−3
miR-422a	0.12	−0.91	0	−2.7
miR-886-5p	0.24	−0.62	0	−2.25
miR-545	0.27	−0.58	0	−2.4
miR-326	0.29	−0.54	0	−2.4
miR-9	0.29	−0.53	0	−2.4
miR-579	0.38	−0.42	0.01	−2.3
miR-124	0.39	−0.41	0.01	−2.3
miR-627	0.42	−0.38	0.01	−2.22
miR-449b	0.5	−0.3	—	—
miR-564	0.65	−0.19	0	−3.62
miR-222^*^	—	—	0	−3.41
miR-15^*^	—	—	0	−3
miR-16-1^*^	—	—	0	−3
Upregulated miRNAs				
miR-7-1^*^	5405.77	3.73	2.01	0.3
miR-126^*^	2486.35	3.4	1.3	0.11
miR-199a-3p	814.45	2.91	10.77	1.03
miR-489	592.42	2.77	7.83	0.89
miR-194	484.64	2.69	6.41	0.81
miR-590-5p	397.96	2.6	5.26	0.72
let-7e	311.16	2.49	4.11	0.61
miR-15b	261.75	2.42	3.46	0.54
miR-19a	245.95	2.39	3.25	0.51
miR-19b	233.54	2.37	3.09	0.49
miR-376c	228.43	2.36	3.02	0.48
miR-324-5p	224.46	2.35	2.97	0.47
miR-374a	218.5	2.34	2.89	0.46
miR-27b	193.3	2.29	2.56	0.41
miR-452	182.34	2.26	2.41	0.38
miR-96	176.66	2.25	2.34	0.37
miR-210	171.29	2.23	2.26	0.35
miR-139-5b	155.02	2.19	2.05	0.31

PBMCs: peripheral blood mononuclear cells; CHC: chronic hepatitis C.

^*^Real-time relative quantification (RQ) values.

**Table 3:** Target miRNAs expression in PBMCs of CHC patients with different HCV genotype and healthy controls.

miRNAs	GT-1	Non-GT-1	Control	*P* value
miR-16	155.065 (10.950)^*^	245.167 (25.742)	13.604 (1.713)	<0.001
>1.713	54	15	24	
≦1.713	12	5	24	
*P* value	0.001			
miR-122	0.0016 (0.0002)	0.0000003 (0)	0.0031 (0.0004)	<0.001
>0.0004	17	0	22	
≦0.0004	54	20	26	
*P* value	<0.001			
miR-193b	0.2175 (0.0070)	0.0068 (0.00076)	0.0033 (0.00013)	<0.001
>0.00013	64	13	24	
≦0.00013	6	7	24	
*P* value	<0.001			
miR-199a	1366.614 (513.816)	95.7169 (33.5522)	479.1835 (9.4969)	<0.001
>9.4969	65	13	24	
≦9.4969	4	7	24	
*P* value	<0.001			
miR-214	159.1452 (0.4719)	677.42 (0.5559)	20.2853 (0.2282)	0.943
>0.2282	41	12	24	
≦0.2282	26	8	24	
*P* value	0.469			
miR-222	3.9360 (0.4564)	2.0039 (0.1394)	0.4898 (0.0103)	<0.001
>0.0103	61	18	23	
≦0.0103	5	2	24	
*P* value	<0.001			
miR-324	49.4397 (2.2015)	21.9596 (4.2781)	9.4064 (0.0524)	
>0.0524	53	16	24	
≦0.0524	12	4	24	
*P* value	0.001			

PBMCs: peripheral blood mononuclear cells; CHC: chronic hepatitis C.

^*^Data were presented by mean (medium).

**Table 4:** The association of the targets miRNAs expression levels and clinical factors (such as ALT, AST, and liver fibrosis) of the CHC patients.

Parameters	miR-193b	miR-199a-3p	miR-122	miR-16	miR-214	miR-222	miR-324-3p
ALT							
<40 U/L	0.169 ± 0.838	777.105 ± 2178.070	0.003 ± 0.010	21.762 ± 69.405	18.220 ± 65.577	0.425 ± 0.954	7.771 ± 39.245
≧40 U/L	0.214 ± 1.464	1113.280 ± 497.265	0.001 ± 0.002	212.344 ± 504.989	54.410 ± 200.481	3.425 ± 11.770	26.781 ± 67.231
*P* value	0.001	0.009	<0.0001	<0.0001	0.235	<0.0001	0.001
AST							
<40 U/L	0.027 ± 0.005	426.223 ± 1115.870	0.003 ± 0.012	13.217 ± 40.283	17.412 ± 64.851	0.496 ± 1.135	11.157 ± 48.112
≧40 U/L	0.174 ± 1.297	882.580 ± 436.044	0.001 ± 0.003	168.314 ± 445.087	45.757 ± 177.139	2.631 ± 10.244	20.214 ± 58.935
*P* value	<0.0001	<0.0001	<0.0001	<0.0001	0.760	<0.0001	0.001
Liver cirrhosis							
No	0.228 ± 1.410	964.452 ± 454.544	0.017 ± 0.122	197.502 ± 490.549	150.749 ± 891.345	2.529 ± 5.821	45.846 ± 209.718
Yes	0.012 ± 0.029	1340.147 ± 440.160	0.001 ± 0.003	109.524 ± 243.190	40.417 ± 79.656	6.452 ± 19.406	34.221 ± 86.321
*P* value	0.221	0.800	0.866	0.996	0.054	0.584	0.508

AST: aspartate aminotransferase; ALT: alanine aminotransferase.

**Table 5:** Multivariate logistic regression analysis of the risk of CHC.

Parameters	Favorable/unfavorable	OR	95% CI	*P* value
Gender	Female/male	0.227	0.080–0.648	0.006
Age	Per year	1.182	1.1062013;1.262	<0.001
BMI	≧27/<27	2.090	0.394–11.074	0.386
Six target miRNAs	High/low	7.202	2.014–25.727	0.002

Six target miRNAs were including miR-16, miR-122, miR-193b, miR-199a-3p, miR-222, and miR-324-3p.

In CHC patients' PBMC, the expression levels of miR-16, miR-122, miR-193b, miR-199a-3p, miR-222, and miR-324-3p were increased and miR-122 expression was decreased compared with healthy controls.
