# Supplementary material for: MicroRNA Expression Profiling in PBMCs: A Potential Diagnostic Biomarker of Chronic Hepatitis C
Source: Dis Markers. 2014 Nov 18;2014:367157. doi: 10.1155/2014/367157 (PMC4255053; doi:10.1155/2014/367157)
Supplement: Supplementary file 1 — In the present study, the expression of miR-122 was not correlated with clinical parameters of CHC, including the viral load (Supplemental Table 1). In addition, we found that those with high levels of expression of miR-193b and miR-199a-3p had an approximately 6.545-fold risk of CHC compared with those with low levels of expression of miR-193b and miR-199a-3p (95% CI =2.180–19.651, p=0.001; Supplemental Table 2). [file 367157.f1.pdf]

Supplemental Table 1. Correlation of target miRNAs expression and clinical parameters in PBMC of CHC patients.

	Age	Hcv RNA	AST	ALT	rGT
miR-193b	r^2 = 0.026	r2 = -0.153	r2 = -0.189	r2 = -0.093	r2 = -0.090
	p = 0.854	*p* = 0.285	*p* = 0.207	*p* = 0.518	*p* = 0.648
miR-199a-3p	r2 = -0.117	r2 = -0.161	r2 = -0.080	r2 = -0.068	r2 = -0.136
	p = 0.417	*p* = 0.265	*p* = 0.600	*p* = 0.639	*p* = 0.491
miR-122	r2 = -0.038	r2 = 0.019	r2 = -0.021	r2= 0.002	r2 = 0.254
	p = 0.790	*p* = 0.895	*p* = 0.889	*p* = 0.987	*p* = 0.192
miR-16	r2 = 0.059	r2 = -0.061	r2 = 0.013	r2 = -0.034	r2 = -0.089
	p = 0.693	*p* = 0.679	*p* = 0.936	*p* = 0.817	*p* = 0.657
miR-214	r2 = 0.050	r2 = -0.058	r2 = 0.279	r2 = 0.241	r2 = 0.001
	p = 0.731	*p* = 0.695	*P* = 0.067	*p* = 0.095	*p* = 0.996
miR-222	r2 = 0.045	r2 = -0.113	r2 = 0.037	r2 = -0.059	r2 = -0.180
	p = 0.763	*p* = 0.445	*P* = 0.814	*p* = 0.692	*p* = 0.369
miR-324-3p	r2 = 0.032	r2 = -0.084	r2 = 0.044	r2 = -0.034	r2= -0.114
	p = 0.830	*p* = 0.570	*P* = 0.779	*p* = 0.820	*p* = 0.571

Supplemental Table 2. Multi-variant logistic regression analysis of the risk of CHC.

Parameters	Favorable/ unfavorable	OR	95% CI	p value
Gender	Female/male	0.302	0.106-0.862	0.025
Age	per year	1.174	1.103-1.249	<0.001
BMI	≧27/<27	2.226	0.407-12.187	0.356
Two targets miRNAs				
	High / low	6.545	2.180-19.651	0.001

Two targets miRNAs were including mir-193b and mir-199a-3p.
